# Divided States of America: Regional Variation in Cardiovascular Health

**DOI:** 10.1161/JAHA.112.006114

**Published:** 2012-12-19

**Authors:** Donna K. Arnett

**Affiliations:** 1 Department of Epidemiology, School of Public Health, University of Alabama at Birmingham, Birmingham, AL (D.K.A.)

**Keywords:** editorials, disparities, health status, heart, regional variation

## Introduction

Approximately one third of the United States adult population has 1 or more types of cardiovascular disease (CVD), and CVD is the underlying cause for about one third of American deaths (2008 data).^1^ CVDs are responsible for more deaths annually than accidents, chronic lower respiratory disease, and cancer combined.^2^ In 2004, the American Heart Association (AHA) set an ambitious goal to reduce the mortality rates of congestive heart disease (CHD) and stroke by 25% by 2010, using 1999 rates as the baseline. The impact of these goals was significant: during the first decade of this century, deaths from CHD and stroke fell by about 30%.^3^ Public health campaigns of primordial, primary, and secondary prevention that concentrated on cardiovascular risk factors likely had considerable influence on mortality rates. Pharmaceutical interventions have dramatically affected risk factors such as cholesterol and blood pressure, with high cholesterol dropping by 25% from baseline and uncontrolled high blood pressure prevalence falling by ≈30%.^3^ Initiatives to help individuals change “lifestyle” factors such as smoking, physical activity, and diet met with mixed success; smoking prevalence has decreased from baseline levels by 16%, whereas trends toward increasing physical activity were more modest.^3^

In the belief that prevention was a key contributor to the success of the 2010 goals, the AHA set a challenging new impact goal for 2020: *to improve the cardiovascular health of all Americans by 20% while reducing deaths from cardiovascular diseases and stroke by 20%*. By developing a new definition of cardiovascular health, goals expanded from only reducing mortality to also positively affecting health factors and health behaviors. The AHA established 7 health factor and behavior categories, including smoking, diet, physical activity, body weight, blood pressure, cholesterol, and glucose, and specified criteria to score individuals and populations as achieving “poor,” “intermediate,” or “ideal” levels of health for metrics in each category. The AHA's goal is to move individuals and the population from poor to ideal CV health in each of the 7 categories. Detailed descriptions of the health metrics and what constitutes each level of health can be found in Lloyd‐Jones et al.^3^

The analysis by Fang and colleagues^4^ in this issue represents one of several recent efforts to use the 7 AHA health factors and behaviors in various post hoc analyses to explore the relationship of ideal cardiovascular health and incident CVD (using ARIC cohort data),^5^ to estimate population‐attributable risk of the factors and behaviors and measure associations and with mortality risk (using National Health and Nutrition Examination Survey [NHANES] data),^6^ and to use recent trends to predict cardiovascular health in 2020 (using NHANES data).^7^ Fang and colleagues used data from the 2009 state‐based Behavioral Risk Factor Surveillance System (BRFSS) and 7 derived AHA‐like measures to estimate ideal, poor, and mean cardiovascular health for each US state and the District of Columbia. With telephone interview data from 356 441 eligible participants, Fang et al detailed self‐reported hypertension, hypercholesterolemia, diabetes, body mass index, smoking status, moderate and vigorous physical activity, and 5 or more servings of fruits and vegetables for each participant. Fang et al concluded that 3.3% of the US population was in ideal cardiovascular health (ie, had ideal health for all 7 metrics), and 9.9% was in poor cardiovascular health (ie, had ideal health for 0 to 2 metrics). Nationally, the average cardiovascular health (ie, number of 7 metrics rated as ideal) was 4.42%. Among the states, the percentage of individuals with ideal cardiovascular health varied from 1.2% (Oklahoma) to 6.9% (District of Columiba). The adjusted prevalence ratio, using median state Illinois as a referent, ranged from 0.38 in Oklahoma to 1.91 in the District of Columbia. The authors concluded that, although higher than previous population estimates,^5–6^ BRFSS data indicate that rate of ideal cardiovascular health in the United States was low. They also observed that cardiovascular health in the United States varies considerably by age, sex, race/ethnicity, and education as well as by state.

The authors propose that their higher‐than‐expected estimates of cardiovascular health may have resulted from a number of design factors: individuals with a reported history of coronary heart disease were excluded, the BRFSS had limited diet data that may not have been a suitable proxy for the equivalent AHA metric, and—most importantly—the BRFSS gathers self‐reported data. They cite findings that indicate self‐reported height and weight tend to be high and low, respectively, when compared with the empirical measures,^8^ and low awareness of hypertension and high cholesterol may have led to low self‐reporting of these conditions.^9^ The authors were quite forthright about the limitations of their self‐reported data, so this point is not raised here as a critique of their efforts. Rather, this post hoc analysis underscores the importance of collecting surveillance data that is up to the task of assessing the cardiovascular health status of each state within the United States, as well as our progress toward the AHA 2020 impact goals. Whenever possible, objective data on health factors and behaviors should be collected in a laboratory or clinic by trained investigators. Of course, smoking, diet, and physical activity behavior data will virtually always be collected by self‐report; strategies for maximizing the accuracy of self‐reported data should be employed, such as those outlined by Newell and colleagues.^10^

**Figure 1.**
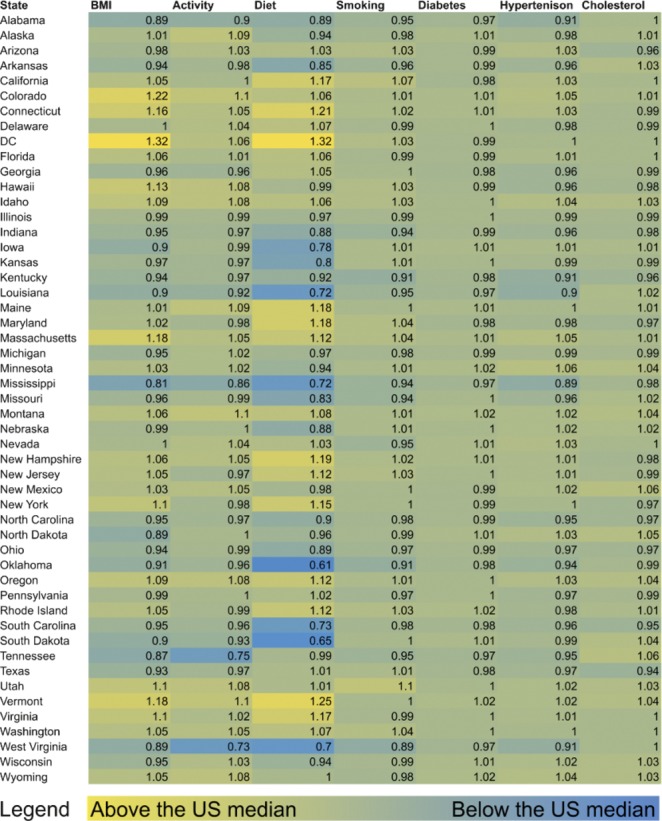
Heat plot (after Fang et al^4^) showing age‐standardized scores of individual cardiovascular health metrics by state (for each metric, calculated as the inverse of the national median percentage score divided by the state percentage score). BMI indicates body mass index (kg/m^2^); activity, ≥150 minutes a week of moderate‐intensity or ≥75 minutes of vigorous‐intensity physical activity; diet, ≥5 fruit or vegetable servings/day; smoking, not smoked ≥100 cigarettes in lifetime or smoked >100 cigarettes in lifetime but not currently smoking; diabetes, never told by doctor, “You have diabetes”; hypertension, never told by health professional, “You have high blood pressure”; cholesterol, never told by health professional, “You have high cholesterol.”

Although the potential biases introduced by self‐reported data may limit the validity of comparisons to external studies, the internal comparisons offered by Fang et al are less likely to be problematic and, in fact, illustrate an expected but critical point: cardiovascular health status across the United States is far from homogeneous. Not only are the integrative measures (ideal, poor, mean cardiovascular health) disparate among states, so are the constituent health factors and behaviors. Moreover, these integrative measures differ substantially across ethnic groups, with disparities present for non‐Hispanic blacks and non‐Hispanic American Indians/Alaska Natives compared with non‐Hispanic whites and non‐Hispanic Asians or Pacific Islanders. The heat plot in Figure 1, a rearticulation of the data in Fang et al's Table S1, provides visual emphasis of this fact. The figure raises a set of questions that beg to be answered, for example: Why are BMI, physical activity, and diet more heterogeneous among the states than smoking, diabetes, hypertension, and cholesterol? What is it about the demographics, culture, and public health efforts (or other characteristics) of Washington, DC, and Vermont that make them contrast so sharply with Oklahoma and West Virginia? These are not merely academic uncertainties. Although it is true that their state‐level characterizations can be used, as Fang and colleagues suggest, “to direct communication initiatives, focus limited resources, and support programmatic plans to improve CV health,” also understanding the forces driving differences among states can serve to improve our ability to create interventions that are customized to specific individuals and populations. A growing body of research suggests that health message framing (ie, whether a health message is conveyed in terms of expected losses or gains from a decision or action), message targeting (ie, adapting a message to a specific group), and message tailoring (ie, adapting a message to an individual) can improve behavioral outcomes.^11–12^ This is particularly relevant in light of the significant disparities in health across educational levels. In all forms of adaptive interventions, however, those designing the messages must know how and why their audiences are segmented and use this knowledge to frame, target, or tailor most effectively. The state‐level characterizations offered by Fang et al represent a crude but critical start to answering these hows and whys.

In conclusion, cardiovascular health metrics and goals provide a unified framework in which public health campaigns can be formulated and assessed; however, regional heterogeneity necessitates that innovative, customized strategies be developed to most effectively improve CV health for specific states and/or other subpopulations.
